# Leveraging neural network models for drug repurposing: a case study on cardiac hypertrophy

**DOI:** 10.1038/s41598-026-68286-z

**Published:** 2026-09-10

**Authors:** Rasmus Magnusson, Markus Johansson, Sepideh Hagvall, Jane Synnergren

**Affiliations:** 1 Systems Biology Research Center, School of Bioscience, University of Skövde, Skövde, Sweden; 2 Department of Biomedical Engineering, Linköping University, Linköping, Sweden; 3 Chief Medical Office, Global Patient Safety, AstraZeneca R&D, Pepparedsleden 1, Mölndal, Sweden; 4 Department of Molecular and Clinical Medicine, Gothenburg University, Institute of Medicine, Sahlgrenska Academy, Gothenburg University, Gothenburg, Sweden

**Keywords:** Cardiology, Computational biology and bioinformatics, Drug discovery

## Abstract

Drug repurposing has emerged as an attractive strategy in contemporary pharmaceutical research, presenting an opportunity to expedite drug discovery, minimize developmental costs, and mitigate risks associated with developing new pharmaceuticals. In this study, we investigated a novel approach based on deep learning of human transcriptomic mechanisms for systematic identification of additional therapeutic potential in preexisting drugs. We trained a composite feedforward neural network model using gene expression data sourced from the ARCHS4 compilation of the GEO, encompassing extensive human datasets. Subsequently, disease-associated gene expression data were generated from our stem cell-derived *in vitro* model of cardiac hypertrophy induced by Endothelin-1 stimulation. These data were employed to identify latent variables associated with genes showing differential expression due to Endothelin-1 stimulation. By examining the differential expression profiles within the model's latent space, we successfully correlated the disease signal with known drug targets found in pharmaceutical compounds cataloged in DrugBank. The model accurately encoded additional disease-related genes beyond the curated gene set, demonstrating its ability to generalize disease associations. Leveraging the model, we identified potential drug candidates, such as lapatinib and amiodarone showing promise in mitigating proBNP concentration associated with cardiac hypertrophy. This study demonstrates the power of deep learning of human transcriptomic mechanisms in swiftly identifying new therapeutic potentials for existing drugs, highlighting the pivotal role of artificial intelligence technologies in accelerating drug development for other complex medical conditions.

**Supplementary Information:**

The online version contains supplementary material available at https://doi.org/10.1038/s41598-026-68286-z.

## Introduction

Drug repurposing involves utilizing approved drugs and failed candidates to target new indications, thereby identifying alternative therapeutic uses for existing medications initially developed for different medical conditions. Exploring existing drugs for their potential therapeutic effects on various diseases or conditions proves economically beneficial^1^, rather than developing entirely new drugs. This process typically integrates advanced computational analysis, high-throughput analyses, and experimental testing to identify potential candidates, particularly those demonstrating promise in targeting molecular pathways associated with new diseases or conditions.

**Fig. 1:**
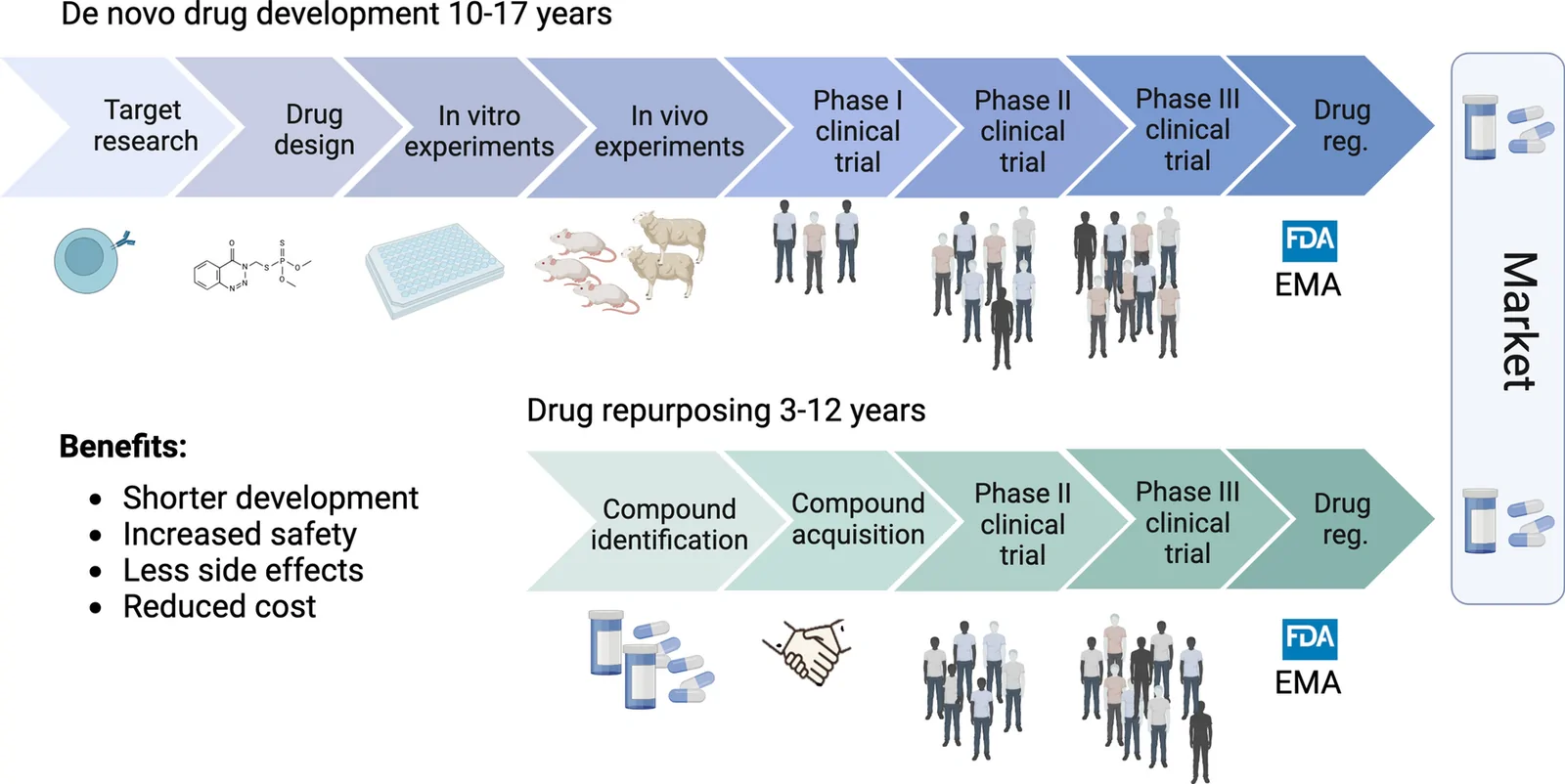
Overview of de novo drug development and indicated in blue are the main steps performed during the traditional drug development process that typically takes between 10-17 years. In green are the main steps of drug development based on drug repurposing outlined, highlighting shorter development, increased safety, less side effects, and reduced cost.

While repurposing drugs for new medical conditions necessitates extensive preclinical and clinical testing to confirm efficacy and safety, it offers substantial advantages compared with *de novo* drug development^2^. This approach facilitates significantly shorter development time as time consuming early steps in the standard drug development process can be omitted or shortened (Fig. 1). For example, since these drugs have already undergone prior safety and efficacy testing, they may benefit from reduced early-phase testing due to existing safety data and prove to be more cost-effective^2^. Repurposed drugs also carry lower risks due to established safety profiles, minimizing unforeseen adverse effects compared to entirely new compounds. These benefits lead to significantly reduced costs and provide possibilities for novel therapies for medical conditions that currently lack efficient treatments. Furthermore, drug repurposing presents opportunities for combined therapies, fostering synergistic treatments for complex diseases^3–6^.

Another important aspect of drug repurposing is the potential for accelerated development of therapies for novel diseases, which is a huge advantage when specific drugs are not yet available or developed. The unpredictable emergence of infectious diseases and the lack of available treatments for rare diseases pose significant global health challenges^7^. The most recent example is the COVID-19 pandemic, which affected many millions of individuals worldwide. During this time, medical practitioners were struggling to find a rapid cure for hospitalized patients to improve the conditions of the patients and minimize outbreak severity. As drug repurposing matches the urgency of finding effective treatments it was applied as an effective strategy in the search for more efficient treatments for COVID-19^8^. One such example is baricitinib, an anti-inflammatory drug approved for rheumatoid arthritis, which was repurposed and authorized for emergency COVID-19 treatment based on encouraging trial results^9^.

**Fig. 2:**
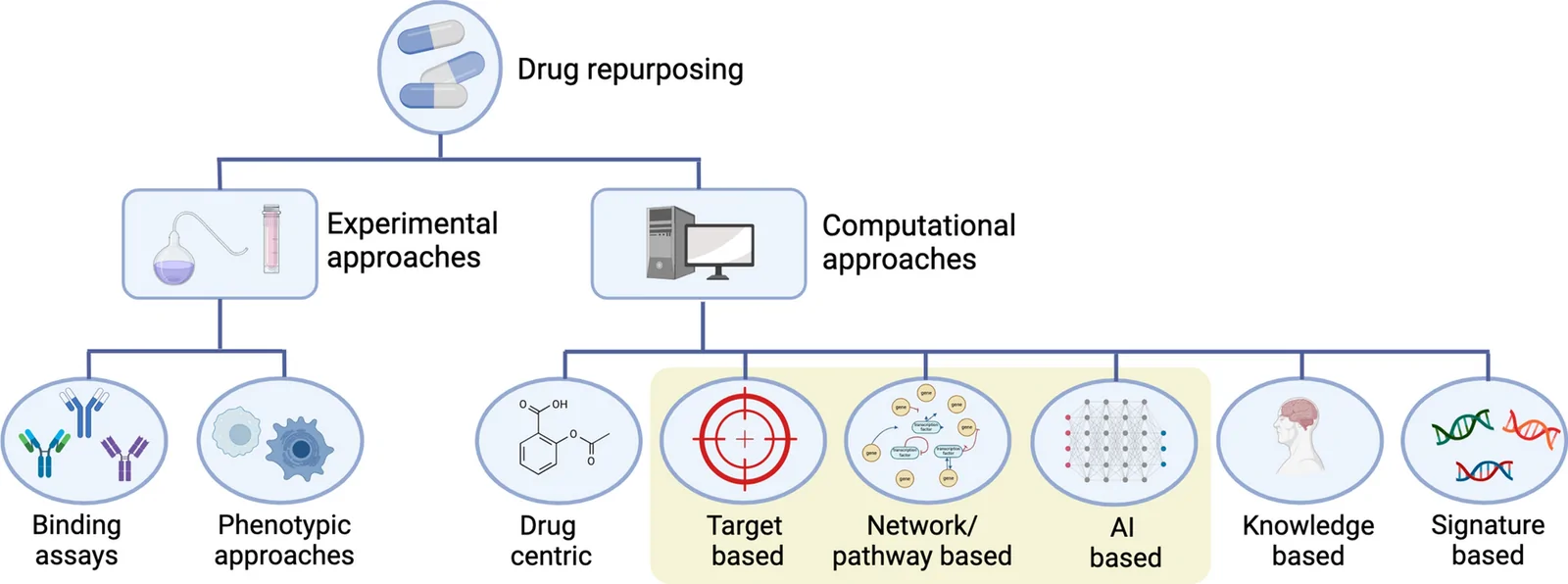
A framework of strategies to perform drug repurposing inspired by Parvathaneni et al.^10^. Both binding assays and phenotypic screening approaches are applied to identify binding interactions of ligands, and identify lead compounds from large compound libraries, and these are classified as *experimental approaches*^6^. The *computational approaches* can be based on different strategies or combinations or strategies. These are generally high-throughput, and thus efficient tools in discovering therapeutic agents for novel indications. In this study we have used a combination of target-based, network-based and AI-based strategies for identification of known drugs that may be candidates for novel indications.

Different strategies are described in the literature to perform drug repurposing^10–14^, and these can be classified into *experimental*- or *computational* approaches. In the experimental approaches binding assays and phenotypic screenings are applied to identify binding interactions of ligands to assay components and identify lead compounds from large compound libraries. The computational approaches include drug centric strategies, target-, network/pathway-, AI-, knowledge-, and signature- based approaches. Importantly, these computational methods accelerate the drug discovery process by effectively utilizing information such as cheminformatics, bioinformatics, network biology and systems biology^11^. In the study presented here we applied a combination of target-, pathway-, and AI-based strategies for drug repurposing (Fig. 2), which demonstrated to be successful in terms of identification of existing drugs for novel indications of cardiac hypertrophy. Based on our result we propose an extension of the framework described by Parvathaneni ^10^, to also include AI based approaches, as highlighted in Fig. 2.

The process of matching known drugs to new diseases is however not straight-forward. There are several reasons for this, including that diseases often involve one or several mechanisms that are seldom fully understood, and that these mechanisms induce changes across the interactome. Nevertheless, the usage of computational biology methods has proven to be an important tool to predict drug repurposing candidates, with numerous success cases^12–14^. This development has recently become of interest from an artificial intelligence and artificial neural network (ANN) modeling perspective^15^. An interesting work by Kuenzi et al.^16^ presents an ANN framework named DrugCell for repurposing drugs. Notably, this framework was constructed by incorporating 2,086 known biological processes from the Gene Ontology database into the structure of the latent space, with the rationale to build an interpretable model structure.

In this study we redesigned the approach used by Kuenzi et al., by building an ANN model with known disease gene sets coded into the latent space. In detail, we used groups of curated gene associations of 1,336 human diseases in the DisGeNET database^17^ to build a composite neural network with a latent space of 1,336 variables, each corresponding to a unique disease set.

This novel drug repurposing approach was then applied to a case study of cardiac hypertrophy to evaluate the performance. Cardiac hypertrophy is a physiological or pathological condition characterized by an increase in the size of the heart muscle, specifically the myocardium^18^. The physiological hypertrophy occurs as a normal and adaptive response to increased workload on the heart, such as during regular exercise^19^ or pregnancy^20^. It leads to an increase in the size of individual cardiomyocytes without significant adverse effects on the heart function. On the other hand, pathological hypertrophy is often associated with chronic stress on the heart and is generally detrimental to the heart function^21^. The heart muscle becomes thicker and stiffer, which impair its ability to contract effectively and pump the blood in the circulatory system^22^. This condition is often associated with detrimental changes in gene expression, signaling pathways, and structural remodeling of the heart. It can develop to a serious maladaptive response that can lead to heart failure, arrhythmias, and other cardiovascular complications. Common causes to pathological hypertrophy include hypertension, aortic stenosis, and cardiomyopathies^23–26^. Notably, even if the cause of the pathological hypertrophy is removed, by the use of existing heart failure therapies, the condition is often retained and most patients do not recover from its diseased condition^27^. Novel therapeutic approaches are needed to help patients to recover and protect them from future cardiac failure.

This study presents a novel approach to drug repurposing through the integration of artificial intelligence methodologies and transcriptomic high-throughput data. By specifically embedding disease-associated gene sets into a composite neural network structure, we demonstrate a capacity to decipher intricate transcriptomic signatures underlying various diseases, with cardiac hypertrophy as a focal case study. This work illuminates the power of neural network learning in comprehending disease mechanisms, offering a transformative approach to swiftly identify therapeutic avenues for complex medical conditions.

## Material and methods

### Transcriptomics data curation

Utilizing gene expression data sourced from the extensive compilation of human RNA-seq experiments in the ARCHS4 database^28^, we initially filtered out pseudo-genes and performed a natural log transformation on the counts of the remaining 27,486 unique genes. To eliminate low-quality entries, such as when very few counts were present in a sample, we excluded samples where over half of the genes showed no detectable expressions. The dataset was partitioned into non-overlapping subsets by systematic subsampling. Specifically, samples were divided by selecting every *n*th observation (with *n = 100*), starting from different offsets, resulting in 10 distinct subsets. This ensured an even distribution of samples across subsets while avoiding overlap. Consequently, we obtained 27,627 curated gene expression profiles, and reserved 8,687 of these for model testing.

### Data analysis and model development

**Fig. 3:**
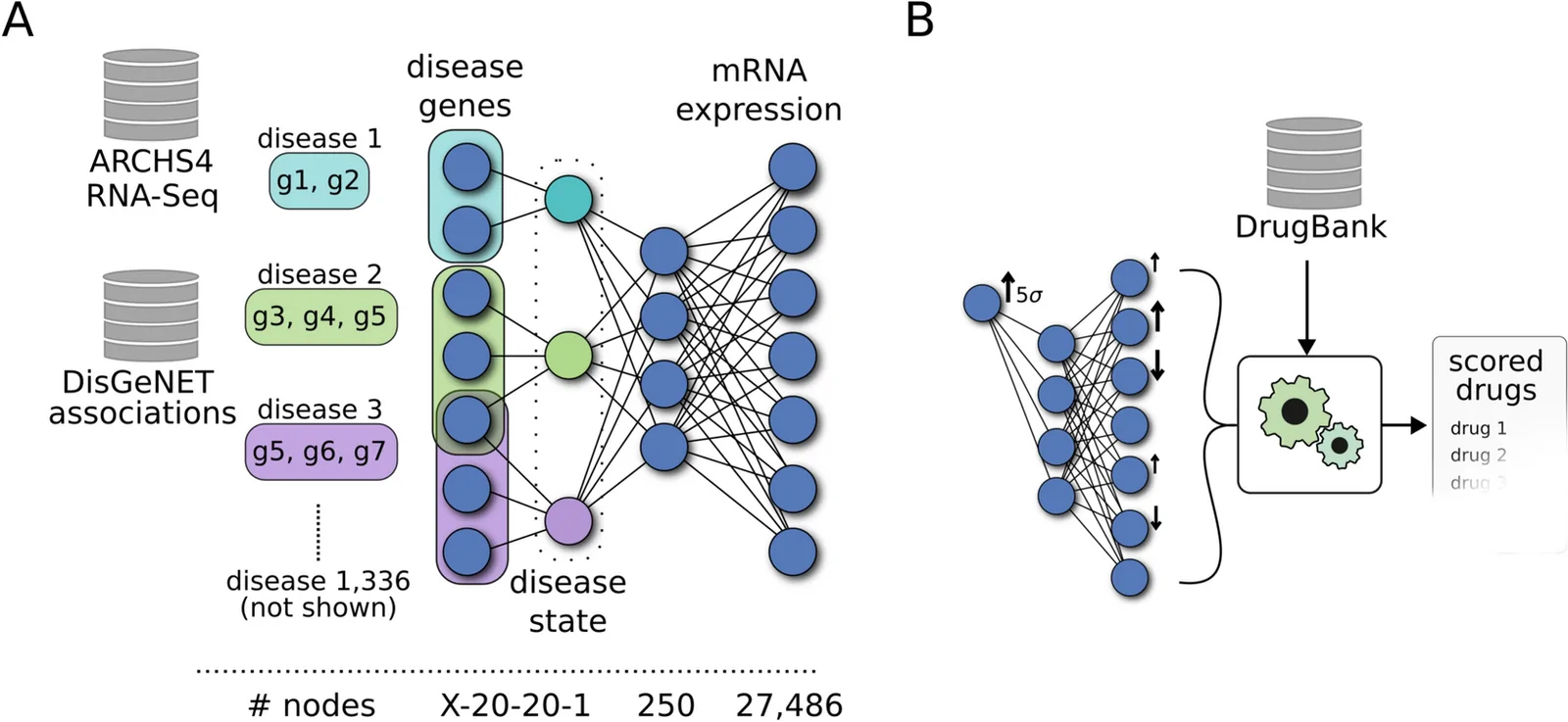
Model design and usage. **A**) We built a feed-forward neural network regression model, using gene expression profiles from the ARCHS4 dataset as input and output. We aimed to embed an explainable latent space, with a layer representing known diseases. To this end, we used gene-disease associations from the DisGeNET, and built 1,336 independent sub-models, i.e. one per disease, all acting as input to the latent space. For a disease with X associated genes, the sub-model would have X inputs, fed to two subsequent layers with 20 nodes each, and finally one single output node. The full model was trained to predict the gene expression of all 27,486 genes. **B**) We perturbed each node in the most compressing hidden layer to associate disease with decoder genes. The decoder output was observed and matched to the target genes of known drugs.

Herein, we used a feed-forward neural network (FFNN) implemented in Keras. The model's architecture aimed to compress gene expression data into a specifically designed latent space by embedding disease-related information. Leveraging the DisGeNET database^17^, we constructed 1,336 individual fully connected feed-forward neural networks, each uniquely representing a disease from DisGeNET. As the DisGeNET includes numerous diseases with a limited set of associated genes, we opted to only include the 1,336 cases with 10 or more associated genes to increase the validity of our results. These disease-specific models were constructed to take the associated gene expressions as input. Inspired by the model design presented in our recently published paper de Weerd et al.^29^, the model featured two hidden layers with 20 nodes each, and culminated in a single node as output. All nodes were implemented with the exponential linear unit as the activation function, similar to our work in^29^. To illustrate, for disease A, we used the n associated genes from the DisGeNET as input and fed them into two hidden layers of 20 nodes each. Subsequently, the 1,336 outputs from the smaller models were concatenated and passed to a fully connected hidden layer comprising 250 nodes. This layer was then connected to an output layer of 27,486 nodes, corresponding to the total number of included genes in our study design. A schematic of the model design can be found in Fig. 3A.

We used a principal component analysis (PCA) to extract features from the latent space encodings of the model. As such, we used the 8,687 test data profiles and calculated the components in the resulting 8,687x1,336 matrix. In order to extract a stronger signal, we chose to independently amplify the data ten-fold along each of the 1,336 components. The rationale for this was that we have previously^29^ found such amplifications to help associate genes with nodes in the latent space. Next, we aimed to study which genes were encoded on each component. Since each component amplification resulted in a continuum of gene associations, we opted to study the respective top-changing genes. In our analysis of the biological processes encoded on each principal component, we individually mapped the 100 genes of each component to a protein-protein interaction (PPI) network. For analyses of biological relevance with respect to the number of top-genes extracted from such an analysis, see our study in^29^. Specifically, we used the STRING and the FunCoup PPI databases, respectively. In gene-gene distance calculations, we used the harmonic mean distance, as this metric is applicable even in the case where two genes are unconnected on a PPI, and the distance is annotated as infinite.

Inspired by the DrugCell method developed by Kuenzi et al. in 2020^16^, this model design facilitated an interpretable latent layer, where each of the 1,336 hidden nodes encoded the gene expression associated with a particular disease. We used the same activation function across all nodes, employing the exponential linear unit (elu), and optimized the models to minimize mean squared error using the Adam algorithm^30^. We trained the model to convergence using batches from the 27,627 gene expression profiles in the training dataset. The model was trained using CPU resources supplied by National Academic Infrastructure for Supercomputing in Sweden (NAISS), consuming <1000 core hours.

### Drug repurposing candidate inference from case-control data

We conducted an analysis using RNA-seq data obtained from our previous study involving induced pluripotent stem cell (iPSC)-derived cardiomyocytes, stimulated with ET-1 (10nM) to induce cardiac hypertrophy, for which the data is publicly available^31^. This dataset served as the basis for detecting significant differences in activations within the hidden layer of our model. We Z-score transformed the activation of each hidden node and selected the node with the highest differential activation as an effect of the ET-1 treatment for further study. In other words, we chose to study the embeddings of the node with the highest relation to ET-1 stimulation. To analyze what genes in the output layer were associated with this hidden node, we employed a 5-standard-deviation activation increase, where the standard deviation was calculated from the validation data in ARCHS4 (Fig. 3B).

These disease-associated genes were then cross-referenced with pharmaceutical compound targets, as referenced in Guney et al.^32^, to rank the drugs. We utilized the gene responses from the 5-standard deviation activation such that we, for each drug, compared the average response in the known drug targets to that of all genes in the decoder. The rationale is that if the drug is associated to the most differentially activated node in the ET-1 dataset, the drug’s target genes should respond to such a perturbation. In equation form, the ranking was made by, for each compound d, calculating the following score

$$score\left(d\right)=\frac{\frac{1}{\left|{G}_{d}\right|}\sum {R}_{g}}{\frac{1}{\left|G\right|}\sum {R}_{g}}$$

Here, G is the set of all genes, and Gd is the set of the genes of known targets of d. The variable Rg is the difference in model output of gene g with respect to the 5-standard deviation activation.

### Analysis of drug properties and characteristics

The list of ranked candidate substances was further manually filtered and substances incompatible with in vitro cell culturing due to limited solubility, high toxicity etc. were excluded. Next, the anticipated impact of these substances on the cardiac hypertrophy signaling pathway was examined applying the Ingenuity Pathway Analysis (IPA) Inginuty^33^. Only the substances that showed inhibition of this signaling pathway and a reduction in a cardiac hypertrophy outcome were considered. The highest-ranked drugs based on their calculated score were selected, one per drug target class, for experimental validation^33^.

The selected drugs include Amiodarone, Binimetinib, Bosentan, Disulfiram, Lapatinib, and Regorafenib. Amiodarone is an antiarrhythmic drug used to treat and prevent conditions such as ventricular tachycardia and atrial fibrillation. Binimetinib is a selective mitogen-activated protein kinase 1/2 inhibitor used to treat a variant of metastatic melanoma. Bosentan, an endothelin receptor antagonist, is used to treat pulmonary arterial hypertension. Disulfiram aids in the treatment of chronic alcoholism by inducing an acute sensitivity to ethanol. Lapatinib, a tyrosine kinase inhibitor, is used in the treatment of breast cancer. Regorafenib, a multi-kinase inhibitor, is utilized in various forms of cancer treatment.

### Cell culture for experimental validation

Human cardiomyocytes (CMs) derived from the hiPSC line ChiPS22 were obtained from Takara Bio (Takara Bio Europe AB). Cells were cryopreserved on day 19 following initiation of differentiation using STEM-CELLBANKER (cat. 11890, Amsbio).

Cryopreserved CMs were thawed in CM medium consisting of Advanced RPMI supplemented with B27 (1×) and GlutaMAX (1×) (Thermo Fisher Scientific), and 10 μM Y-27632 (cat. Y0503, Sigma-Aldrich). Cells were plated at a density of 3.75 × 10^5^ cells per well in 24-well plates pre-coated with fibronectin (5 μg/cm^2^; cat. F0895, Sigma-Aldrich).

**Fig. 4:**
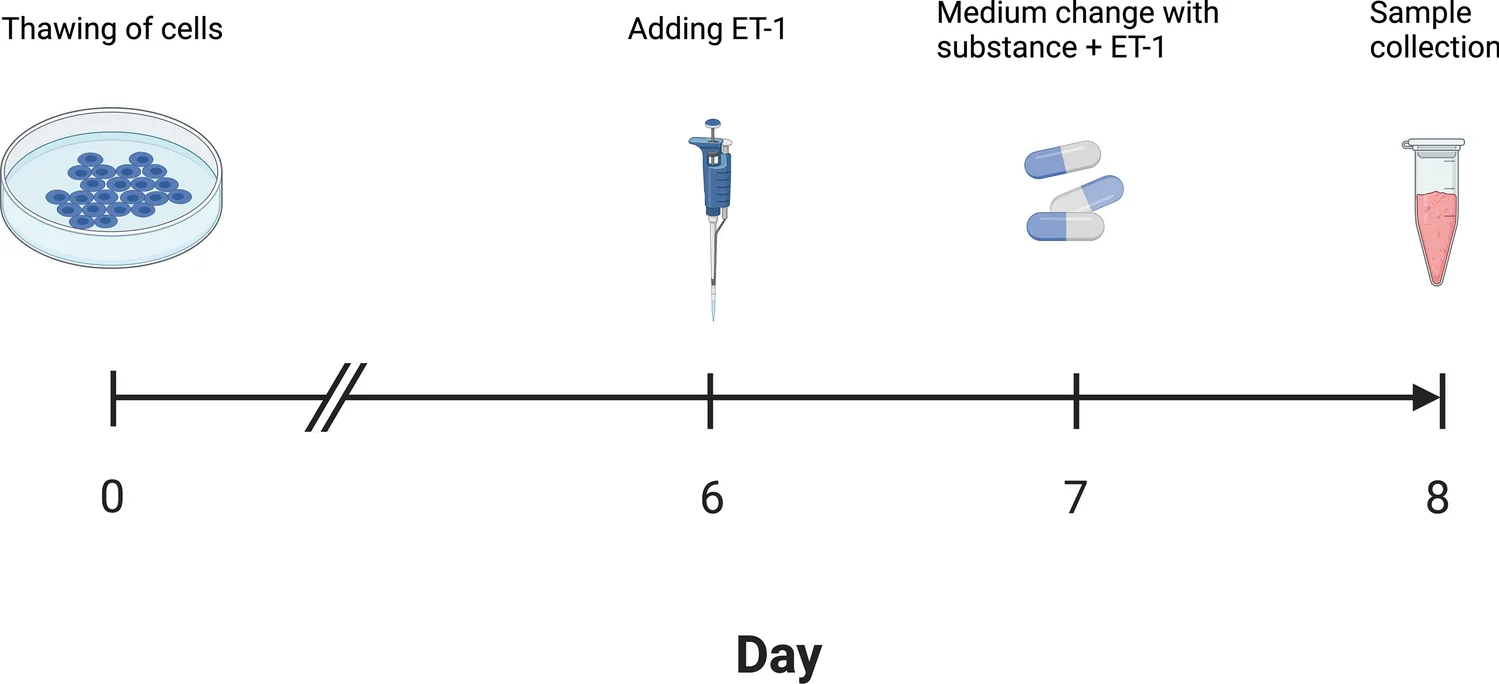
Overview of the experimental design. The hiPSC-CMs were thawed and cultured for 6 days before ET-1 (10nM) was added to the cells. On day 7, the medium was changed, and a new dose of ET-1 was added. On day 8, the medium was collected and stored for later analysis.

Twenty-four hours after thawing, the medium was replaced with CM medium without Y-27632, and cells were allowed to recover for a total of 6 days prior to experimental treatment with endothelin-1 (ET-1). Culture medium (0.4 ml/cm^2^) was refreshed every second day. For hypertrophy induction, ET-1 (cat. E7764, Sigma-Aldrich) 10nM was dissolved in DMSO and added to the culture medium. After 24 h of ET-1 treatment, the medium was replaced with fresh CM medium containing ET-1 in combination with the indicated compounds. The choice of solvent for amiodarone, binimetinib, bosentan, disulfiram, lapatinib, and regorafenib was based on their physicochemical properties. Notably, amiodarone exhibited higher solubility in methanol than in DMSO and was therefore prepared in methanol, whereas all other compounds were dissolved in DMSO. Cells were treated with ET-1 in combination with the respective compounds for 24 h prior to sample collection (Fig. 4). Control cells received equivalent volumes of DMSO or methanol. All experiments were performed in at least three independent replicates. The overall study design is summarized in Fig. 4.

### Enzyme-Linked Immunosorbent Assay (ELISA)

Conditioned media was collected from all the samples 48h after ET-1 was added to the media, and subsequently 24h after the potential hypertrophy inhibitors were added. The media was centrifuged (5000x g, 5 min) and the supernatant was collected and stored at -80° C for subsequent analysis. The concentration of proBNP in the samples was analyzed using ELISA kits (EHPRONPPB, Thermo Fisher Scientific) according to the manufacturer's instructions. Statistical analyses of proBNP concentrations were performed using a paired Student’s t-test. Statistical significance was defined as p < 0.05. Data are presented as mean ±SEM for n=4 independent experiments, with the exception of the 5uM lapatinib group (n=3). Significance levels are indicated as follows: *p < 0.05, **p < 0.01, and ***p < 0.001.

## Results

**Fig. 5:**
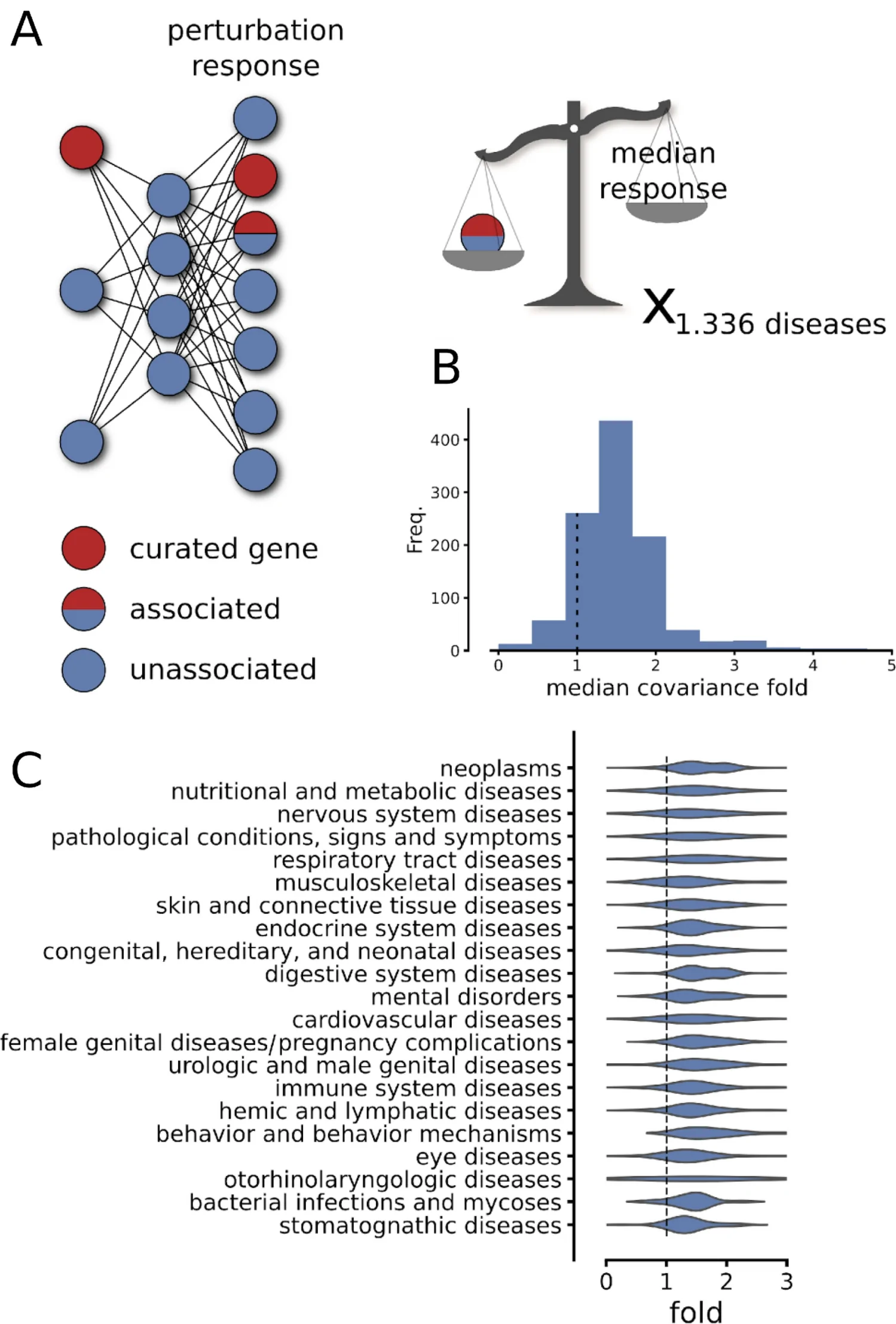
The model training embedded relevant disease structures. **A**) To test how the model generalized in terms of capturing disease mechanisms, a set of disease-gene associations of lesser confidence and unseen by the model was used. For each disease-embedding in the latent space, we recorded how a perturbation of 5 standard deviations changed these associated genes in relation to the median change in the predicted expression. **B**) The median of the response of the associated genes was divided by the overall median response, which is referred to as the covariance fold. Shown is a histogram of these 1,336 folds. Note that the distribution mean is above the expected value of 1. **C**) By stratifying the results shown in **B**) into disease classes, we found a clear signal across all disease types.

Expanding upon recent advances that integrate prior biological knowledge into deep learning models, our investigation aimed to determine if incorporating disease-gene associations within a feedforward neural network (FFNN) could predict specific drug candidates. Initially, we constructed a FFNN utilizing 1,336 disease gene sets as input (Fig. 3A). Finally, leveraging the model (Fig. 5), we identified potential pharmaceutical candidate drugs, focusing on cardiac hypertrophy as a case study. Experimental validation of these results showed a significant reduction in proBNP levels in an in vitro hypertrophy disease model following treatment with amiodarone and lapatinib. The reduction was in similar levels as for the ET-1 receptor antagonist bosentan, which was included as a positive control, indicating their potential therapeutic impact.

### A set of 1,336 disease-specific variables can accurately predict the transcriptome

We aimed to integrate biological a priori knowledge into our model by incorporating sets of disease-related genes for predicting the expression values of the human transcriptome. Utilizing gene-disease associations from DisGeNET, we constructed a FFNN with an interpretable latent space. Specifically, we created individual small FFNN for each gene set, compressing the expression pattern to a single variable representing a disease state. Each FFNN consisted of two hidden layers with 20 nodes each. In total, we employed 1,336 disease states and connected their outputs to a fully connected FFNN (see Fig. 3a). The entire model was optimized to predict the counts of approximately 27,000 human genes from the ARCHS4 database^28^.

We tested the model’s ability to predict gene expression data by applying it to ARCHS4 gene expression profiles that were kept apart from the training data. We found the model to predict new data well, with a median Spearman’s correlation coefficient rho = 0.95. Testing to what extent the mean expression value correlated with the validation data profiles, we observed a median Spearman correlation coefficient of 0.83. Thus, the model could accurately predict a complete gene expression profile from 1,336 hidden variables that were specifically designed to hold information of gene expressions of corresponding diseases.

### Additional disease-associated genes became encoded in relevant latent variables

We next sought to test to what extent the model encodings had biological relevance and opted to analyze a disease association gene set that contained more genes but with lower certainty. In detail, all disease-gene associations registered in the DisGeNET but not part of the curated set that was used to design our model were downloaded and analyzed. We performed a sensitivity analysis on the decoder output with respect to each hidden node in the disease-specific hidden layer by, to each node, adding 5 standard deviations to the mean node activation, and by subsequently observing the value changes in the output layer (Fig. 5A). We found the changes of the genes that were exclusively found in the lower confidence gene-disease annotation list to have a significantly (Mann-Whitney u p < 0.05) higher value than the rest of the genes in 53% of the diseases (expected 5% under the null hypothesis, Fig. 5B-C). These results support that the model has captured relevant disease genes. Notably, the median response of those genes was higher than the expected value of 1 in 88% of all comparisons, suggesting that the number of cases where the null hypothesis is rejected would increase considerably with statistical power.

### Extracting learned biologically relevant patterns from the model latent space

Several researchers, including our group, have previously demonstrated how FFNNs applied to transcriptomic data can learn meaningful representations of cellular biology^34–36^. To investigate the biological relevance of this model’s embedding and extract novel information, we continued our analysis by searching for biologically relevant patterns. In detail, we analyzed the principal components (PCs) of the validation data in the compressed space, such that each PC was individually amplified with a factor 10. These perturbed data were subsequently passed through the decoder, and the top 100 changing genes per PC were extracted. We hypothesized that genes that are associated to the same disease have a higher level of interaction than randomly selected genes. Thus, the internal relation between each gene set of 100 genes was then analyzed with respect to the protein-protein interaction networks in STRINGdb and in FunCoup. Results show that the top PCs correspond to gene sets that were significantly closer on the protein-protein interaction maps than what would be expected under the null hypothesis, i.e. that the genes were unrelated. In fact, the first 10 PC gene sets had a harmonic mean distance that on average was 27% (STRINGdb) and 36% (FunCoup) of the monte-carlo estimated distance of randomly sampled genes. In other words, the latent space of the FFNN appeared to have learnt known biological patterns.

### The differential activation of hidden nodes in case-control data can be interpreted as a novel approach to disease annotation analysis

In this study, the FFNN model was specifically designed to have an interpretable latent space, with each hidden node corresponding to one disease in DisGeNET. Notably, this presents the opportunity to use the model as a gene set annotation tool, where the profiles of case-control gene expression data can be compressed and the nodes in the latent space tested for differential activation. Since each node was designed to represent a specific disease in the DisGeNET, the differential activation is interpretable as an annotation of interest.

To evaluate the model in an applied disease scenario, we utilized RNA-Seq data of endothelin-1 (ET-1) stimulated hiPSC-dervied cardiomyocytes (CMs), which we have previously demonstrated to be a model of cardiac hypertrophy^31,37^. ET-1 stimulated samples were compared to untreated control samples, a Z-score transformation of the differentially activated nodes of the latent space was performed. When analyzing the highest ranked nodes, several relevant diseases e.g. diastolic heart failure and left ventricular hypertrophy were identified. We noted that there was a substantial degree of genes overlapping between the highest ranked terms (mean pairwise odds ratio=14.1, geometric mean of p= 0.0008), with at least one gene overlapping in all sets except the fourth top term, Parkinson disease (PD), suggesting a homogenous disease signal.

The PD activation is not intuitive for a cardiac hypertrophy model and was therefore further investigated. While gene-level overlap with other top terms was absent, pathway-level inspection of the PD-associated decoder genes indicated enrichment for mitochondrial function, oxidative stress, proteostasis, and MAPK components, which are engaged by ET-1 in cardiomyocytes^38–45^. We therefore interpret this activation as a shared stress/mitochondrial program rather than PD-specific biology.

### Matching hypertrophy-induced differential activation in the latent space to known drug targets reveals new avenues for existing drugs

To investigate if the cardiac hypertrophy dysregulation patterns learned in the latent space could be used to predict candidates for drug repurposing, the top differentially activated node activation was increased with a factor of 5 standard deviations, as calculated in the validation data, and the changes in the output layer of the decoder were recorded. These gene changes were matched against known drug targets, as presented in^32^ and a ranked list of drug repurposing candidates was extracted (Suppl. Table 1).

The ranked list of drugs does not indicate whether they are predicted to alleviate or exacerbate cardiac hypertrophy. It also does not indicate whether the drugs are suitable for treating cardiac hypertrophy. Because the initial ranking was direction-agnostic (magnitude only), high scores could reflect compounds that either counteract or reinforce the ET-1–associated signature. Disulfiram, which ranked highly by the unsigned score, increased proBNP in vitro (discussed in section "ProBNP protein concentration after treatment with candidate drugs"), illustrating this limitation.

For example, nitrate compounds, such as nitrous acid and pentaerythritol tetranitrate, received the highest score. However, this type of substance may be unsuitable for treatment of cardiac hypertrophy as it can increase outflow tract obstruction by decreasing preload and ventricular volumes^46^. We therefore emphasize the need for empirical validation and, in future analyses, suggest incorporate direction-aware scoring to prioritize predicted antagonists of the disease signature. Histamine receptor agonists, such as betahistidine and histamine also showed high scoring. Analysis of these substances in Ingenuity Pathway Analysis (IPA) software (QIAGEN Inc., Redwood City, CA, USA, https://digitalinsights.qiagen.com/IPA, accessed april 2021) showed that they had a reverse predicted effect on hypertrophy, rendering them inadequate as a treatment alternative (Suppl. Fig. 4). Beta-blockers were also found to have relatively high scores with Sotalol showing the top score from this drug group (Suppl. Fig. 5). However, Sotalol has been shown to worsen symptoms of decompensated heart failure^47^.

To exclude inadequate drug candidates from the list of results, a manual selection was performed. Only substances with high potential to reduce the concentration of the cardiac hypertrophy biomarker, proBNP, in our developed in vitro disease model^31,37,48^ were selected for experimental validation. The final list of six candidates that was chosen for in vitro validation is shown in Table 1.

**Table 1.** The six candidate drugs that were selected for in vitro validation to evaluate their potential therapeutic effect on cardiac hypertrophy.

Potential drug candidates			
**Drug**	**Score**	**Target**	**Notes**
Bosentan	3,0	ET receptor antagonist	
Lapatinib	2,9	Tyrosine kinase inhibitor	Cancer drug
Disulfiram	2,5	Alcohol dehydrogenase inhibitor, Dopamine beta-hydroxylase inhibitor	Treatment for chronic alcohol abuse
Amiodarone	2,3	Potassium and sodium channel inhibitor	Anti-arrhythmic
Regorafenib	1,5	Kinase inhibitor	Cancer drug
Binimetinib	0,6	MEK1/2 inhibitor	Cancer drug
*Note:*			

**Fig. 6:**
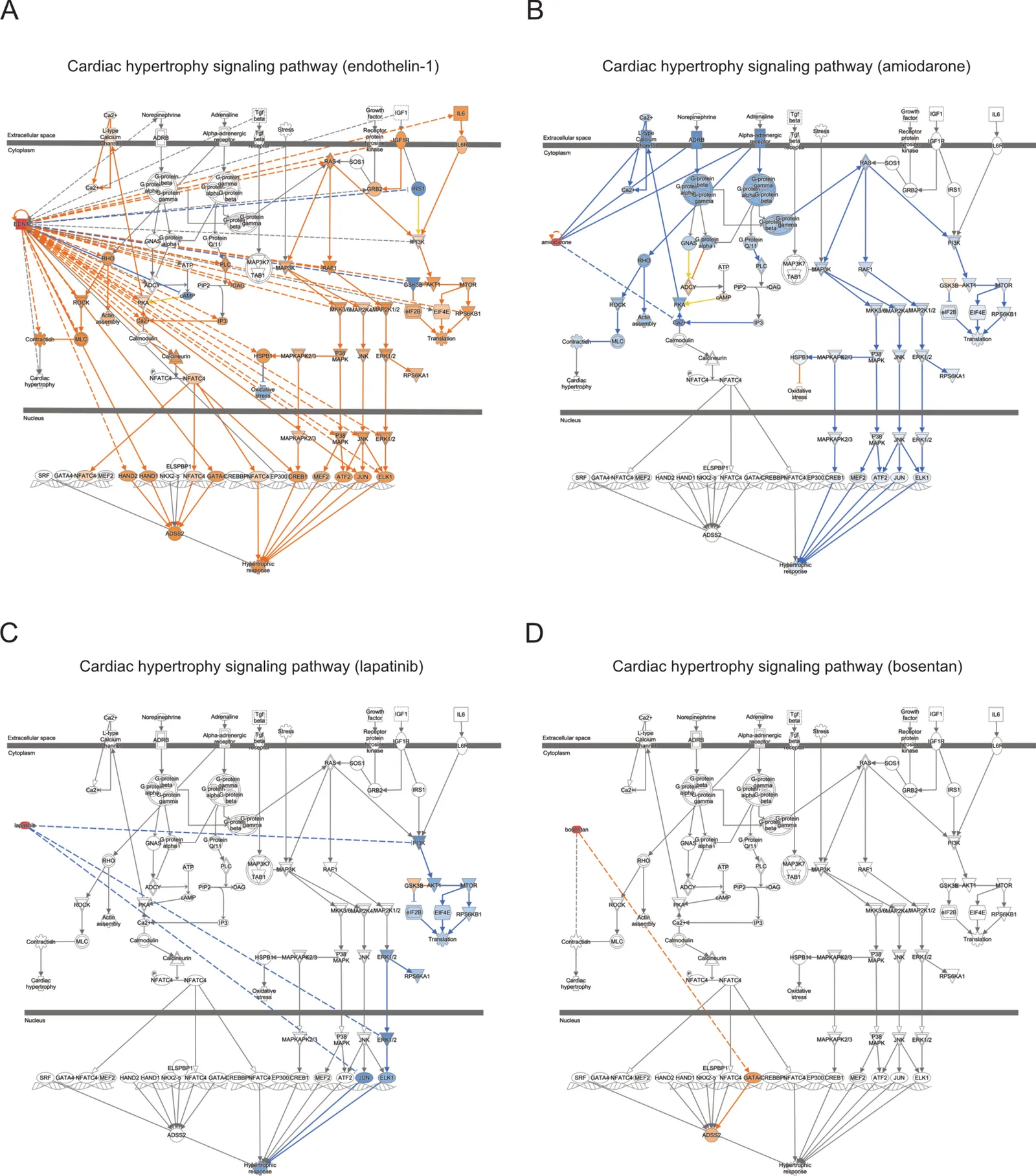
Cardiac hypertrophy signaling pathway. An overview of the signaling pathways that are involved in cardiac hypertrophy and how **A**) endothelin-1, **B**) amiodarone, **C**) lapatinib, and **D**) bosentan affect the signaling. Orange color indicates an increase in expression, and blue color indicates a decrease in expression.

Figure 6 presents an overview of the cardiac hypertrophy signaling pathway and how it is influenced by ET-1, amiodarone, lapatinib, and bosentan. While ET-1 was predicted to increase the hypertrophy response, as expected, both lapatinib and amiodarone were predicted to reduce it.

### ProBNP protein concentration after treatment with candidate drugs

**Fig. 7:**
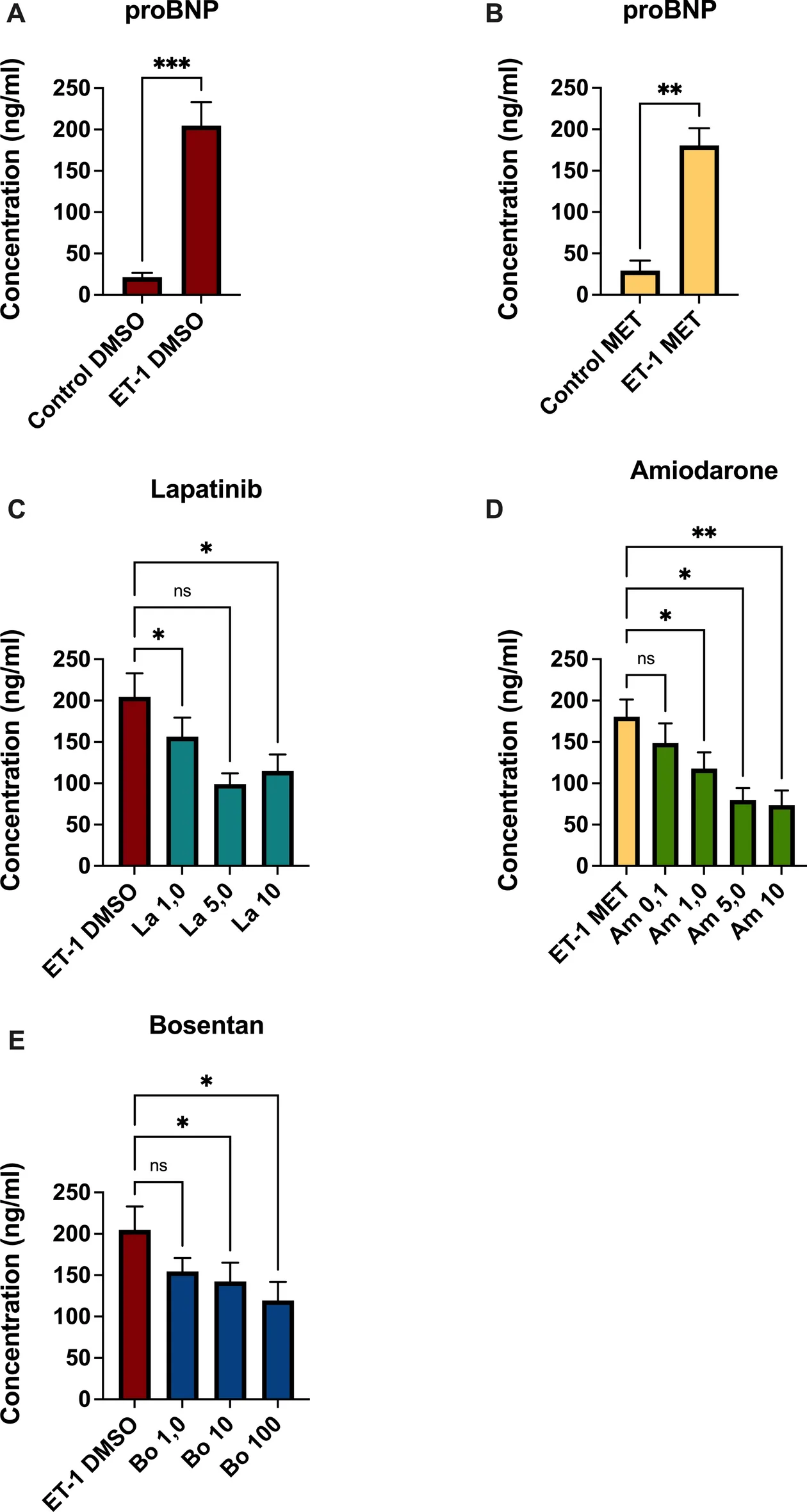
proBNP concentration analysis. Graphs showing the concentration of proBNP in the culture media, concentration in ng/ml, A) Control vs ET-1 (DMSO), B) Control vs ET-1 (Methanol), C) ET-1 (DMSO) vs Lapatinib (1,5,10µM), D) ET-1 (Methanol) vs Amiodarone (0.1, 1, 5 and 10µM) E), ET-1 (DMSO) vs Bosentan (1, 10 and 100µM). SEM is given as error bars n=4, except Lapatinib 5µM (n=3). Significance is defined as *p<0.05, **p<0.01, ***p<0.001, ns=not significant.

The concentration of proBNP, a well-known biomarker for hypertrophy, was analyzed in our established in vitro model of hypertrophy to investigate the potential of the selected candidate drugs to reduce its concentration. Fig. 4 shows an overview of the experimental validation setup. ET-1 stimulation of the CMs for 48h significantly increased the concentration of proBNP in the media for both DMSO (FC = 10.2, p = 0.0002) and methanol controls (FC = 7.8, p = 0.007) (Fig. 7 a-b). ET-1 stimulation for 24h followed by ET-1 stimulation and Lapatinib treatment (1.0 and 10.0µM) for 24h significantly reduced proBNP concentration compared to only ET-1 treatment. At 5.0µM there was a decrease but did not reach statistical significance due to three instead of four replicates for 5.0µM (p = 0.06) (Fig. 7c). In the acute hiPSC-CM assay, lapatinib reduced proBNP at the tested concentrations and exposure without qualitative signs of cytotoxicity on brightfield inspection. As quantitative viability was not measured in this experiment, we cannot exclude a cytotoxic contribution, and interpret this decrease as a short-term disease-biology signal.

Treatment with Amiodarone decreased the proBNP concentration in a dose-dependent manner. At 5.0 and 10.0µM the reduction in proBNP concentration was over 100ng/ml (Fig. 7d). The ET-1 receptor antagonist, Bosentan, significantly reduced the proBNP concentration at 10.0 and 100µM (62ng/ml and 85ng/ml respectively) but did not reach the level of reduction as Amiodarone did (Fig. 7e).

Treatment with disulfiram, regorafenib and binimetinib showed no significant reduction of proBNP concentration (Suppl. Fig. 1). Of note, disulfiram increased proBNP levels above ET-1 alone at both concentrations tested (1 nM and 100 nM), with mean values of ~700 ng/ml and ~510 ng/ml, respectively, compared with ~430 ng/ml for ET-1 controls (Suppl. Fig. 1). These data indicate a potential pro-hypertrophic effect of disulfiram in our acute ET-1–stimulated hiPSC-CM model.

A limitation in this study is the lack of quantitative viability data, but available qualitative observations are shown in Suppl. Fig. 2-Suppl. Fig. 3.

## Discussion

Over the past decade the cost of drug development has surged while annual approvals have not increased proportionally, driven by scientific complexity, regulatory demands, and personalized medicine efforts^49–52^. Many candidates fail for efficacy and safety reasons, and clinical trials are costly and lengthy^53,54^. Against this backdrop, drug repurposing has grown in relevance, with a substantial fraction of approvals arising from new indications for existing agents^2,6,10,53,55,56^. Repurposing can shorten development timelines, leverage known pharmacology, and reduce cost and animal use^49,55–57^. This study particularly focuses on the scientific problem of systematically identifying repurposing candidates by integrating an interpretable neural network with transcriptomic data.

### Model design, interpretability, and learned biology

We specifically addressed interpretability by encoding curated disease–gene associations directly into the latent layer of a composite feed-forward neural network trained on large human RNA-seq compendia. This design, conceptually related to interpretable architectures such as DrugCell yet distinct in its disease-aligned latent basis, allowed direct differential activation analysis from case–control data and mapping of the most responsive latent node to decoder genes and drug targets^16,34–36^. The model reconstructed transcriptomes with high fidelity and captured biologically coherent structure in the latent space, including enrichment of protein–protein interaction proximity and generalization to additional, lower-confidence disease–gene annotations (with appropriate multiple hypothesis considerations), indicating that disease-relevant patterns were learned beyond the curated inputs.

Biological mechanisms of identified drugs in the hypertrophy context applying the model to ET-1–stimulated hiPSC-derived cardiomyocytes, we identified latent activations aligned with cardiac phenotypes and used decoder perturbations to prioritize drugs with targets most responsive to the ET-1–associated latent signal. The validated compounds map onto known hypertrophy pathways. Lapatinib inhibits ERBB family signaling (EGFR/ERBB2), which intersects with MAPK/ERK and PI3K–AKT cascades involved in cardiomyocyte stress adaptation and growth; its experimentally observed reduction of proBNP is compatible with attenuation of upstream receptor tyrosine kinase inputs to these axes in the acute setting^58,59^. Lapatinib has a recognized cardiac risk profile, including asymptomatic LVEF decline and rare heart failure events, particularly in oncology regimens and with concomitant cardiotoxic therapies. ERBB family signaling contributes to cardiomyocyte stress adaptation; its inhibition may therefore carry on-target cardiac risks. In our acute hiPSC-CM assay, lapatinib reduced proBNP without overt cytotoxicity at the tested concentrations and exposure. However, short-term biomarker changes do not establish safety. We interpret these data as a disease-biology signal indicating that ERBB-linked pathways modulate the ET-1 hypertrophy response. Any translational consideration would require confirmation across orthogonal hypertrophy endpoints (e.g., cell area, fetal gene program), longer exposures, and concentration ranges aligned to clinically achievable unbound levels, together with rigorous cardiac safety evaluation.

Amiodarone modulates multiple ion channels and exerts anti-adrenergic effects. By influencing intracellular calcium handling and downstream calcineurin/NFAT and CaMKII signaling pathways, it can attenuate hypertrophic transcriptional programs, consistent with the observed dose-dependent reduction in proBNP expression^58,59^. In addition to our candidate drugs, we also included bosentan as a positive control. Bosentan acts as a dual endothelin receptor antagonist with higher affinity for ET_A, a key mediator of Gq/11-dependent hypertrophic signaling. Its partial reduction of proBNP serves as a pathway-proximal positive control within the tolerated concentration range^60,61^. The incomplete suppression of ET-1–induced effects observed with bosentan may be explained by suboptimal compound concentration under the experimental conditions; however, further dose escalation was not feasible in the hypertrophy model due to induction of cytotoxicity. Together, these findings support that the model’s disease-aligned latent representation captures established hypertrophic biology and can prioritize mechanistically plausible modulators.

### Consistency between AI predictions and experimental outcomes

The initial ranking nominated drugs whose targets were predicted to be most affected by perturbing the ET-1–responsive latent node. Two of the top candidates, lapatinib and amiodarone, reduced proBNP in vitro, indicating concordance between predicted mechanistic leverage points and phenotypic attenuation. In contrast, disulfiram did not produce a beneficial effect in our assay and increased proBNP, consistent with the possibility that its predicted effects align with (rather than oppose) the ET-1–responsive signature. In addition, disulfiram’s known activities (e.g., ALDH inhibition and redox/catecholamine modulation) may exacerbate stress responses in ET-1–treated cardiomyocytes. This underscores the importance of directionality in computational ranking and the necessity of experimental validation. We explicitly acknowledge this limitation in our pipeline description and results, and we discuss that factors such as pathway context, off-target activities, and acute versus chronic effects can influence directionality, reinforcing the need to pair computational prioritization with bench testing.

### Advantages over traditional high-throughput screening

Relative to traditional high-throughput screening, our approach offers three practical advantages within the scope of this study. First, interpretability: latent variables aligned to disease gene sets enable disease-specific annotations from case–control transcriptomes and transparent mapping to drug targets rather than treating the model as a black box^16,34–36^. Second, transcriptome-wide context: by reconstructing the full expression profile, the decoder provides a systems-level view of predicted on- and off-pathway effects connected to known disease mechanisms and drug targets. Third, scalability: after training on large public datasets^28^, the same model can be applied rapidly to new case–control inputs to triage candidates prior to experimental work, potentially reducing downstream screening burden while enriching for mechanistically grounded hits.

### Limitations and future directions

Our analysis uses single-node perturbations to ascribe effects, which may conflate correlated disease states; alternative attribution strategies could refine causality^29,62,63^. Heterogeneity in public RNA-seq data and the acute exposure design are additional constraints; confirmation with orthogonal hypertrophy endpoints (e.g., proBNP alongside complementary markers), chronic dosing, and independent datasets will strengthen translation^64–68^. Finally, while the positive controls and validated compounds support the pipeline, broader pharmacology and safety assessments—particularly for agents with known cardiac liabilities—are outside the present scope and will be necessary for any translational consideration^49,55,56^.

## Conclusions

We demonstrate that a disease-aligned, interpretable neural network trained on human transcriptomes can connect case–control disease signals to drug targets and surface mechanistically plausible repurposing candidates. In an ET-1 hypertrophy model, two prioritized agents reduced proBNP *in vitro*, supporting the biological relevance of the learned latent representation. This framework complements existing discovery approaches by combining interpretability, transcriptome-wide context, and scalability, and it provides a basis for mechanistically guided triage prior to focused experimental validation.

## Supplementary Information

Supplementary Information 1.

Supplementary Information 2.

Supplementary Information 3.

Supplementary Information 4.

Supplementary Information 5.

Supplementary Information 6.

Supplementary Information 7.

## Data Availability

RNA-seq data from the cardiac hypertrophy model can be accessed at: https://www.ebi.ac.uk/biostudies/arrayexpress/studies/E-MTAB-11030.

## Code availability

The code for the model can be accessed at https://github.com/rasma774/composite_disease_model.
