# Supplementary material for: Leveraging neural network models for drug repurposing: a case study on cardiac hypertrophy
Source: Sci Rep. 2026 Sep 10;16:28333. doi: 10.1038/s41598-026-68286-z (PMC13562686; doi:10.1038/s41598-026-68286-z)
Supplement: Supplementary file 1 [file 41598_2026_68286_MOESM1_ESM.pdf]

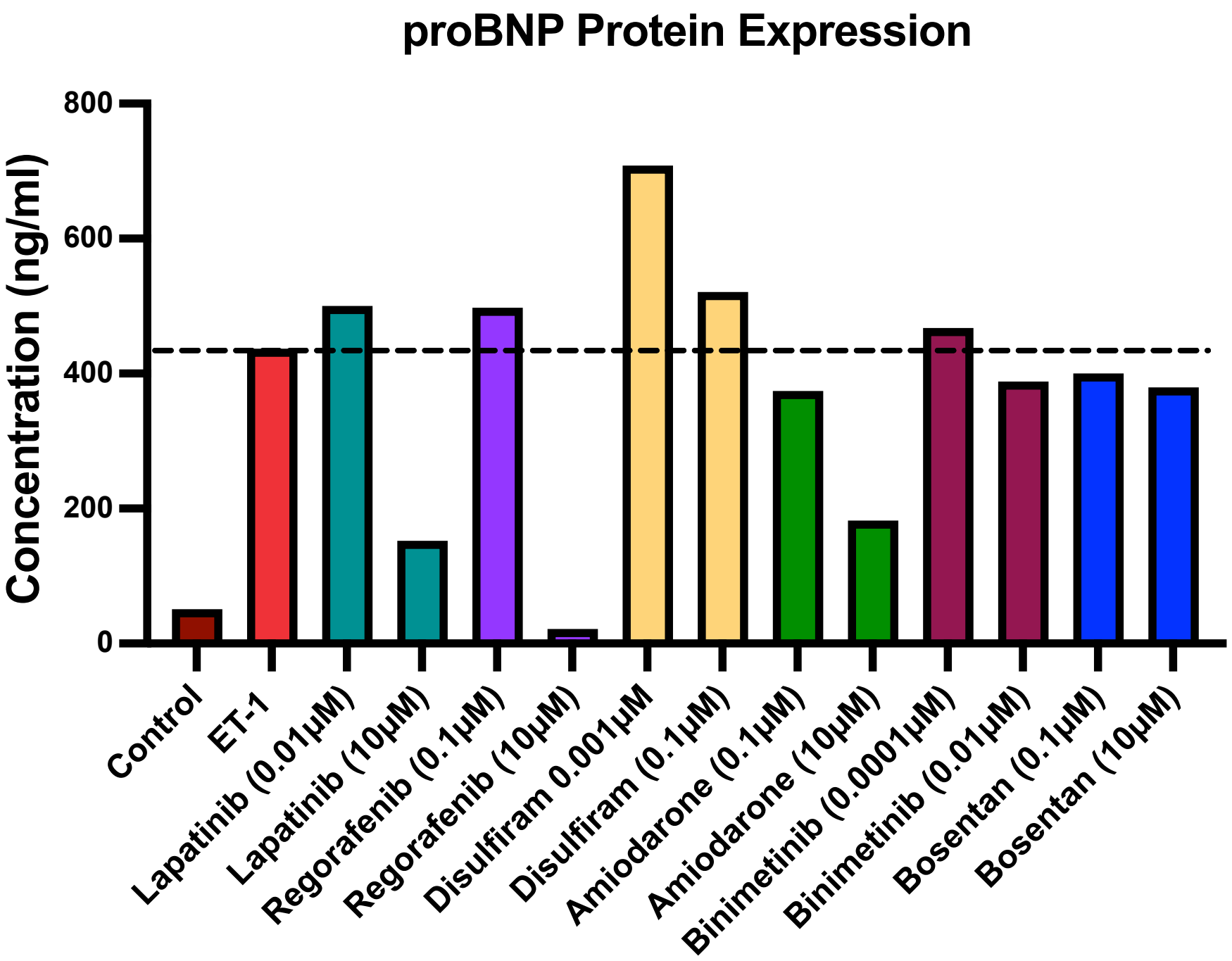
proBNP Protein Expression
Concentration (ng/ml)
800
600
400
200
0
Control
ET-1
Lapatinib (0.01μM)
Lapatinib (10μM)
Regorafenib (0.1μM)
Regorafenib (10μM)
Disulfiram 0.001μM
Disulfiram (0.1μM)
Amiodarone (0.1μM)
Amiodarone (10μM)
Binimetinib (0.0001μM)
Binimetinib (0.01μM)
Bosentan (0.1μM)
Bosentan (10μM)
