# Supplementary material for: Leveraging neural network models for drug repurposing: a case study on cardiac hypertrophy
Source: Sci Rep. 2026 Sep 10;16:28333. doi: 10.1038/s41598-026-68286-z (PMC13562686; doi:10.1038/s41598-026-68286-z)
Supplement: Supplementary file 2 [file 41598_2026_68286_MOESM2_ESM.pdf]

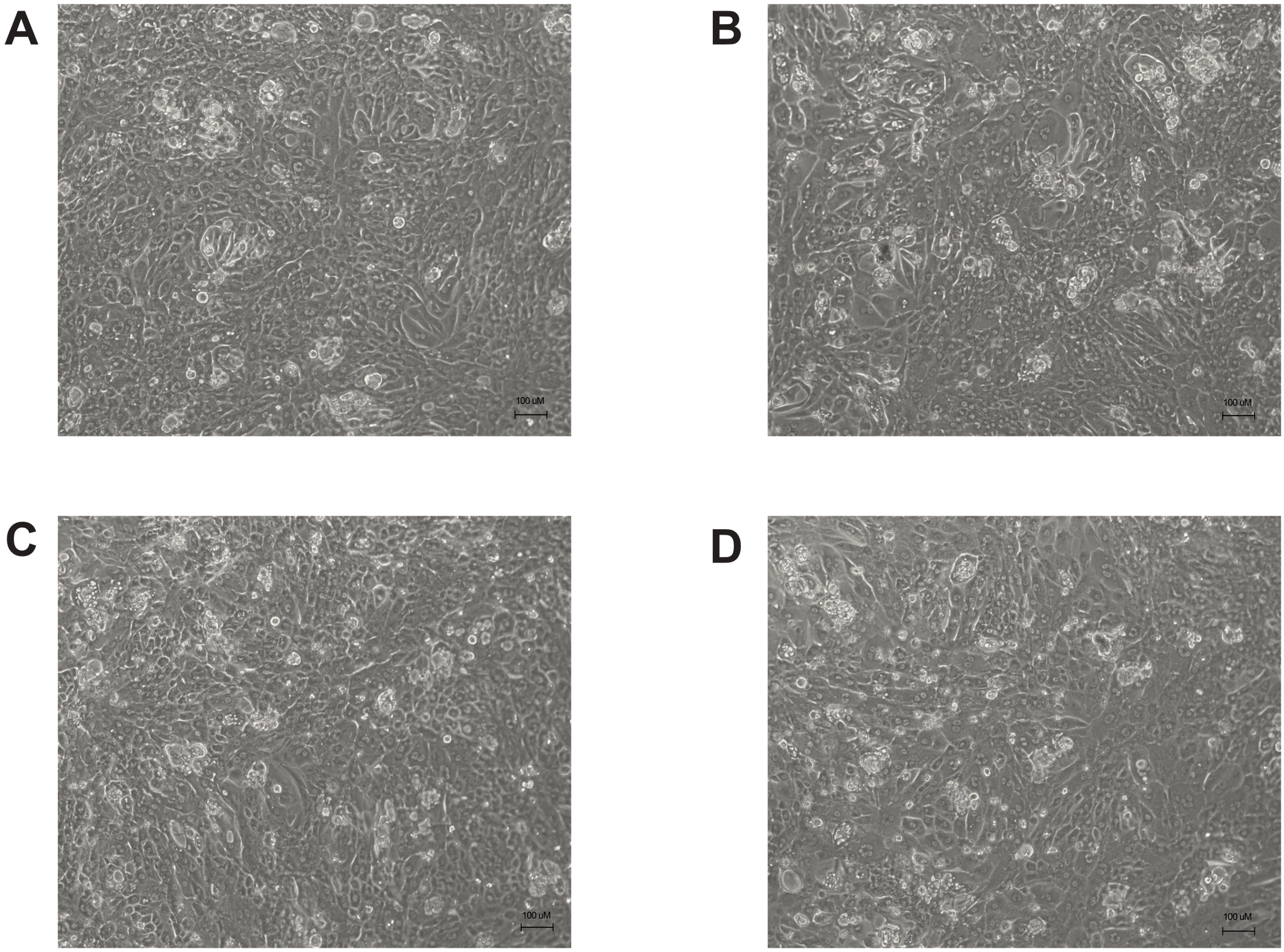
A
B
C
D
100 uM
100 uM
100 uM
100 uM
