# Supplementary material for: Leveraging neural network models for drug repurposing: a case study on cardiac hypertrophy
Source: Sci Rep. 2026 Sep 10;16:28333. doi: 10.1038/s41598-026-68286-z (PMC13562686; doi:10.1038/s41598-026-68286-z)
Supplement: Supplementary file 3 [file 41598_2026_68286_MOESM3_ESM.pdf]

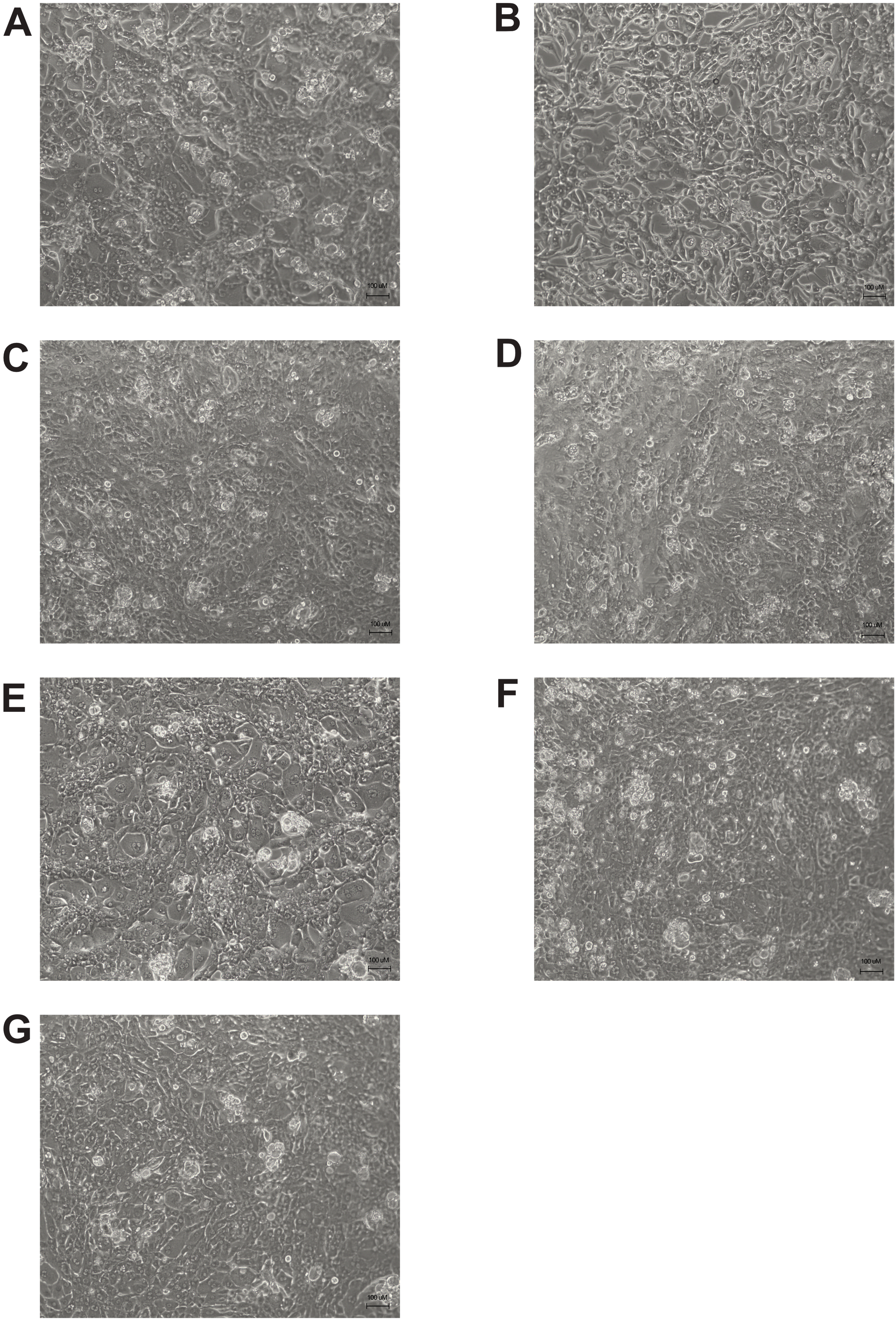
A
100 uM
B
100 uM
C
100 uM
D
100 uM
E
100 uM
F
100 uM
G
100 uM
