# Supplementary material for: Leveraging neural network models for drug repurposing: a case study on cardiac hypertrophy
Source: Sci Rep. 2026 Sep 10;16:28333. doi: 10.1038/s41598-026-68286-z (PMC13562686; doi:10.1038/s41598-026-68286-z)
Supplement: Supplementary file 5 [file 41598_2026_68286_MOESM5_ESM.pdf]

Cardiac Hypertrophy Signaling (Enhanced)

Cardiac hypertrophy is an abnormal enlargement of the heart muscle originating from an increase in cell size of myocytes.
It is initially an adaptive response to normalize wall tension and maintain cardiac output, but if the stress persists
it becomes a pathological event which can lead to heart failure.

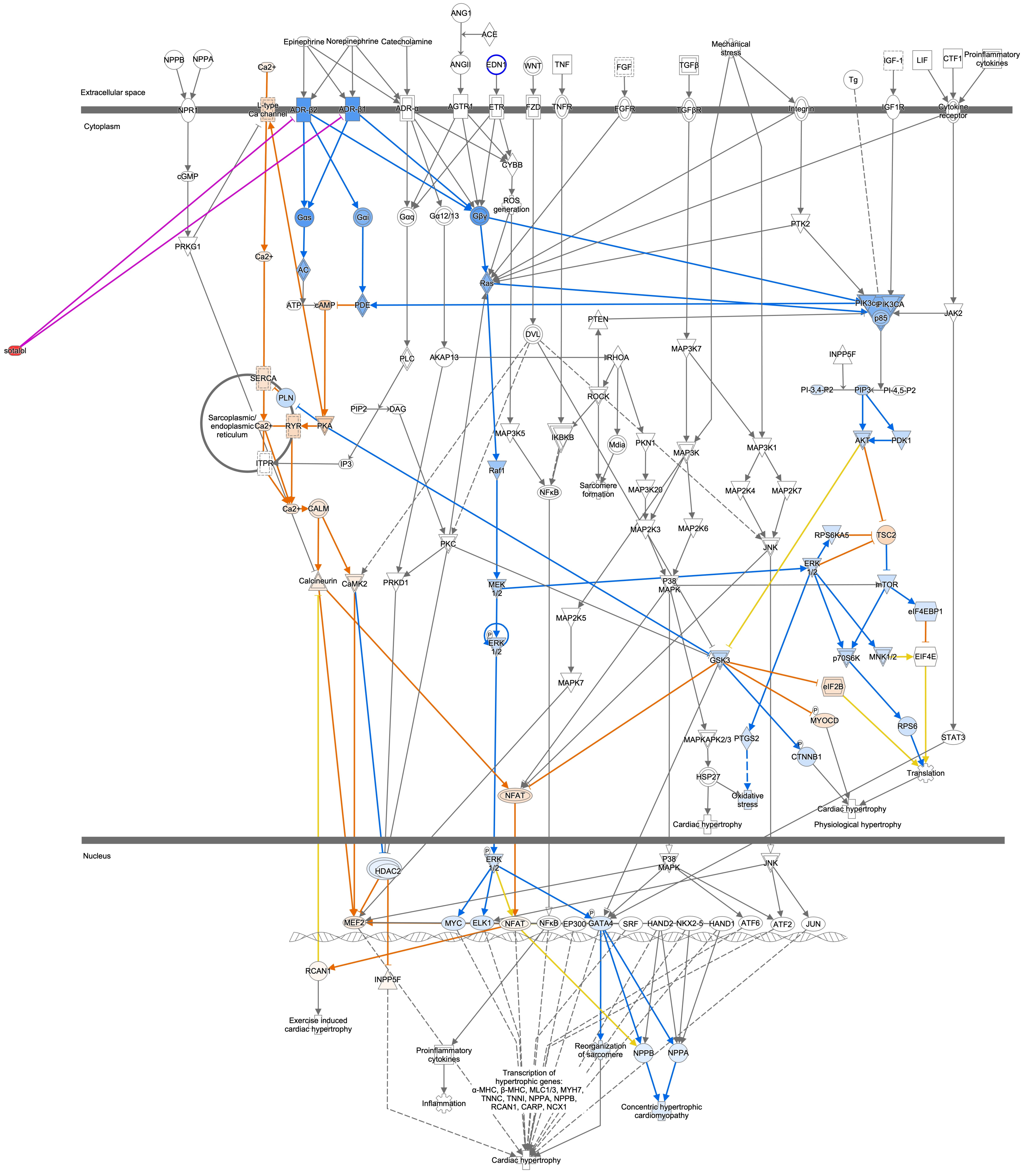

© 2000-2021 QIAGEN. All rights reserved.
