# Supplementary material for: Leveraging neural network models for drug repurposing: a case study on cardiac hypertrophy
Source: Sci Rep. 2026 Sep 10;16:28333. doi: 10.1038/s41598-026-68286-z (PMC13562686; doi:10.1038/s41598-026-68286-z)
Supplement: Supplementary file 6 [file 41598_2026_68286_MOESM7_ESM.docx]

Supplementary Figure legends.

**Suppl. Fig 1.** proBNP concentration analysis of all substances tested. Initial screening to investigate which substances that reduced the proBNP concentration. The y-axis represents the concentration in ng/ml, and x-axis shows the different treatment groups. The dashed line represents the mean ET-1 control value.

**Suppl. Fig. 2.** Brightfield images captured immediately before sample collection. Magnification for all images is 10x. A) Amiodarone 1.0uM B) Amiodarone 10uM C) Lapatinib 0.1uM D) Lapatinib 1.0uM E) Lapatinib 10uM F) Bosentan 10uM G) Bosentan 100uM

**Suppl. Fig. 3.** Brightfield images captured immediately before sample collection. Magnification for all images is 10x. A) Control (DMSO) B) Control (Methanol) C) ET-1 (DMSO) D) ET-1 (Methanol)

**Suppl. Fig. 4.** The influence of Betahistine on the cardiac hypertrophy signaling pathway predicted using the Ingenuity Pathway Analysis.

**Suppl. Fig. 5.** The influence of Sotalol on the cardiac hypertrophy signaling pathway predicted using the Ingenuity Pathway Analysis.
